# High prevalence of overweight among adolescents in Ho Chi Minh City, Vietnam

**DOI:** 10.1186/1471-2458-13-141

**Published:** 2013-02-15

**Authors:** Phuong Van Ngoc Nguyen, Tang Kim Hong, Truong Hoang, Dung The Nguyen, Annie R Robert

**Affiliations:** 1 Pham Ngoc Thach Medical University, 86/2 Thanh Thai Street, District 1, Ho Chi Minh City, Vietnam; 2 Université catholique de Louvain - SSS/IREC/EPID , Institut de Recherche Expérimentale et Clinique, Clos Chapelle-aux-Champs 30 – UCL B1.30.13, B1200, Brussels, Belgium; 3 Research division EPID, Epidemiology and Biostatistics, School of Public Health, Brussels, Belgium

**Keywords:** Adolescent overweight, Ho Chi Minh City, IOTF definition, Obesity, Prevalence, Socioeconomic, Vietnam

## Abstract

**Background:**

Two previous surveys conducted in Ho Chi Minh City revealed an increasing prevalence of overweight and obese adolescents, from 5.9% in 2002 to 11.7% in 2004. From 2004 to 2010, the government set up and implemented health promotion programs to promote physical activity and good nutritional habits in order to prevent overweight and obesity in children and adolescents. Our study aimed to estimate the prevalence of overweight and obesity among adolescents in urban areas of Ho Chi Minh City in 2010.

**Methods:**

A representative sample of 1,989 students aged 11–14 years was selected using a multistage cluster sampling method. 23 schools were randomly selected from the full list of all public junior high schools. In each selected school, 2 classes were chosen at random and all students from the class were examined. Age- and sex-adjusted overweight and obesity were defined using International Obesity Taskforce cut-offs.

**Results:**

The prevalences of overweight and obesity were 17.8% and 3.2%, respectively. Prevalences of overweight and obesity were significantly higher in boys (22%, 5.4% ) than in girls (13.3%, 1.3%, p<0.001) and higher in children from districts with a high economic level (20.5% , 3.8% ) than in those from districts with a low economic level (12.1%, 3.8%, p<0.001). Additionally, children living in wealthier families were more overweight and obese than those living in less wealthy families. When using WHO cutoffs, the overall prevalences of overweight and obesity reached 19.6% and 7.9%, respectively.

**Conclusion:**

Our study’s findings suggest that the prevalence of overweight and obesity among secondary school students remains high, especially among boys living in wealthier families. Public health programs should therefore be developed or improved in order to promote good eating habits and physical activity among youth in HCMC.

## Background

The prevalence of overweight and obesity among children and adolescents is gradually increasing worldwide. Although the highest prevalence rates of childhood obesity are observed in developed countries, obesity is also increasing in developing countries [1]. In East Asia and South East Asia, rapid urbanization and socio-economic development combined with changes in eating habits and in physical activities have led to an increase in obesity in adults, as well as in children [2].

Vietnam is a country located in South East Asia with 86,927,700 inhabitants [3]. Over the past decade, eating habits of Vietnamese people changed substantially [4]. There was a rapid and sustained reduction in prevalence of underweight (weight-for-age z-score < −2) from 33.8% in 2000 to 18.9% in 2009 among children aged under 5 [5]. At the same time, prevalence of overweight and obesity increased rapidly, especially among children and adolescents and in urban areas. In 2005, the National Survey on overweight and obesity showed that the prevalence among adults in Vietnam was about 16.3% in urban areas. The prevalence among children less than 5 years almost doubled in 3 years, rising from 3.2% in 2002 to 6.3% in 2005 [6].

In Vietnam, many studies about overweight and obesity have been conducted in children less than 10 years of age. Few studies have looked at this issue among adolescents (11–14 years of age). Obesity in adolescence substantially increases the risk of and its associated health deficiencies in adulthood. Additionally, obesity in adulthood is a high risk factor for cardiovascular diseases and chronic diseases, such as hyperlipidaemia, hyperinsulinemia, hypertension, and early atherosclerosis [7,8].

Ho Chi Minh City (HCMC) is the largest city in Vietnam. According to a study conducted in 2004, which used International Obesity Taskforce (IOTF) cutoffs, the prevalences of overweight and obese adolescents in HCMC were 11.7% and 2.1%, respectively. A cross-sectional study in 2002 reported a prevalence of overweight and obesity among adolescents of HCMC of 5.9% and 0.7% [9]. These results demonstrate the high increase of overweight and obesity in adolescents from HCMC over two years.

From 2004 to 2010, the government set up and implemented health promotion programs to promote physical activity and good nutritional habits to prevent overweight and obesity in children and adolescents [10,11]. Our study was conducted in order to estimate the prevalence of overweight and obesity among adolescents in Ho Chi Minh City in 2010. The findings aim to contribute to the understanding of adolescents’ nutritional status in urban areas of Vietnam and we hope to offer elements for developing strategies for prevention and control among adolescents of Ho Chi Minh City.

## Methods

### Study design

We conducted a cross-sectional survey among grades 6–9 (11–14 years) of HCMC junior high schools in 2010. It is estimated that about 92% of adolescents living in HCMC are attending secondary high schools [12]. HCMC is administratively divided into 24 districts: 5 rural districts, 5 suburban (districts numbers 2, 7, 9, and 12), and 14 urban districts [13]. This study was conducted in urban districts only.

Urban districts number 1, 3, 5, and 10 are classified as wealthy districts by the National Department of Statistics. There are about one million inhabitants (20% of the urban population of HCMC) including 54,505 students in grades 6–9 attending the 45 public junior high schools (data provided by the City Department of Education for the year 2009–2010). We randomly selected 15 of these 45 schools, using a probability proportional to the size of the target population (the number of students in grades 6–9 within each school).

In the remaining ten urban districts, there are 138,589 students in grades 6–9 attending the 99 public junior high schools. We randomly selected 8 of these 99 schools, using also a probability proportional to the size of the target population. Due to the random process, no schools were selected in district 6, the Phu Nhuan district, and the Binh Tan district, while two schools were selected in the Tan Phu district. One school was selected per district for the six remaining districts.

In each selected school, we randomly selected a class in grades 6–7 and one class in grades 8–9 using a uniform probability model. All adolescents of a selected class were invited to participate to the study.

For such a multistage cluster sampling method, a sample size of 1,530 students was required to have a precision of 0.05 on the estimated prevalence, with a correction for clustering effect of 2.1 and with a prior prevalence of 12% in wealthy districts and 2% in less wealthy districts [9]. With an expected missing data rate of around 25%, 2000 students needed to be enrolled in the study. Based on the detailed data of the HCMC Department of Education, we computed a mean number of 45 students per class. With 46 classes (23 schools), the expected number of students was 2,070: 1,350 in wealthy districts and 720 in less wealthy districts.

When combining data for wealthy and non wealthy districts, weights of 0.17 and 0.83 corrected for the lower proportion of students in wealthy districts (28%) than in non wealthy districts (72%).

### Data collection

Self-administered questionnaires were used to collect information from students and their parents. The researcher and trained interviewers gave students standardized instructions for filling out the questionnaires, which were completed in the classroom in 2 h. Interviewers were present during questionnaires completion to answer questions as necessary. The questionnaire for students included a pubertal questionnaire concerning the date of their first menstruation (for girls) and the date of voice change (for boys), as well as Tanner photographs illustrating five stages of pubertal development for male genitalia, male pubic hair, and female breasts.

The questionnaire for parents included height and weight information, socio-demographic data and household information such as family size, education and occupation, whether they owned domestic assets (telephone, radio, video cassette player, CD system, DVD player, air conditioner, refrigerator, computer, gas stove, microwave, bicycle, motorbike, car, TV). The questionnaire for parents was brought home by the student and returned to school on the next day.

### Measures

#### Anthropometric measures

Students were weighed while wearing light clothes without shoes using a Tanita electronic scale (Tanita BF 571, Tanita Corporation, Japan). Heights were measured using a suspended Microtoise tape with standard methodology. Measurements were recorded by a trained worker. Body mass index (BMI) was calculated as the weight in kilograms divided by the square of the height in meters.

#### Assessment of the pubertal stage

For each student, pubertal stage was assessed as pre-pubescent or pubescent according to the WHO definition [14]. For girls, the pre-pubescent corresponds to breasts at stage 1, and no menarche; pubescent corresponds to breasts at stage 2 or more and post-menarche. For boys, prepubescent corresponds to genitalia at stage 2 or less, and pubescent corresponds to genitalia at stage 3 or more.

### Data analysis

Analyses were conducted using STATA 10 (STATA Corporation, College Station, TX, USA, 2008). In order to compare our prevalences to previous surveys conducted in HCMC, the IOTF cutoffs were used [15,16]. However, BMI z-scores were also calculated using the WHO 2007 reference of BMI-for-age in children aged 5–19 years [17].

A household wealth index was calculated as the first principal component of the variance-covariance matrix of 14 dummy variables coding for ownership of 14 assets. This wealth index was categorized into 5 subgroups according to quintiles. Prevalence ratios were used as comparison indices. Trends across categories were tested using Cochran χ2 tests. Trends across ages were tested using linear slope tests. The statistical significance level was set to 0.05.

### Ethical considerations

Standards of ethics in studies conducted in Vietnam were respected. The study protocol was approved by the Health Service of HCMC and by the Department of Education and Training of HCMC. The study was a collaboration between Pham Ngoc Thach Medical University and the Catholic University of Louvain (Brussels, Belgium). The protocol was approved by both universities. Informed consent was obtained from parents, students, and from principals with standard assurances of confidentiality.

## Results

2,051 students participated in this study. Anthropometric measurements were gathered from 2,050 students. Family forms were missing (not returned) for 61 students, yielding 1,989 cases available for analyses.

In this study, the proportion of boys was 47.6%. Mean weight, height, BMI, and BMI z-score for each age group and gender are presented in Table 1. Boys were significantly heavier and taller than girls within each age group. As expected, the mean weight, height and BMI progressively increased with age in boys and in girls. However, the mean BMI z-score decreased with age in both genders; in boys, its decrease was 0.30 ± 0.04 per year of age, two times higher than the decrease observed among girls 0.14 ± 0.03 per year of age (p < 0.002).

**Table 1:** Anthropometric measurements by age and gender of 1989 pupils from Ho Chi Minh City

**Characteristics**		**≤ 11 yrs**	**12 yrs**	**13 yrs**	**≥ 14 yrs**	**Total**
		**(Mean± SD)**	**(Mean± SD)**	**(Mean± SD)**	**(Mean± SD)**	
**N**	Boys	193	227	326	201	947
	Girls	190	301	332	219	1042
**Weight (kg)**	Boys	42 ± 12	46 ± 11	51 ± 12	53 ± 12	48 ± 12
	Girls	40 ± 9	44 ± 8	47 ± 8	46 ± 8	45 ± 8
**Height (cm)**	Boys	145 ± 8	152 ± 8	158 ± 8	163 ± 8	155 ± 10
	Girls	146 ± 7	152 ± 6	153 ± 6	154 ± 6	152 ± 7
**BMI**	Boys	19.8 ± 3.8	19.9 ± 3.7	20.0 ± 4.0	19.7 ± 3.6	19.9 ± 3.8
**(kg/m**^**2**^**)**	Girls	18.5 ± 3.1	19.1 ± 2.7	19.9 ± 3.0	19.5 ± 2.9	19.3 ± 3.0
**BMI z-score**	Boys	0.74 ± 1.42	0.50 ± 1.35	0.22 ± 1.42	−0.17 ± 1.31	0.31 ± 1.42
	Girls	0.13 ± 1.20	0.11 ± 1.03	0.09 ± 1.07	−0.31 ± 1.05	0.02 ± 1.09

*BMI z-score: z-score based on WHO 2007 reference values*[17]*.*

Table 2 summarizes the anthropometric parameters by age and school location of 1989 pupils from HCMC. The mean weight, height, and BMI were higher in pupils studying in schools located in a wealthy district than in pupils studying in schools located in a less wealthy district. As shown in Table 2, BMI z-score decreased significantly with age in both wealthy and less wealthy districts but scores were higher in wealthy districts than in less wealthy districts, at each age.

**Table 2:** Anthropometric measurements by age and school location of 1989 pupils from Ho Chi Minh City

**Characteristics**		**≤ 11 yrs**	**12 yrs**	**13 yrs**	**≥ 14 yrs**	**Total**
		**(**$\bar{x}$**± SD)**	**(**$\bar{x}$**± SD)**	**(**$\bar{x}$**± SD)**	**(**$\bar{x}$**± SD)**	
**n**	Wealthy districts (n^*^)	261 (7)	328 (5)	454 (12)	236 (6)	1279 (30)
	Less wealthy districts (n^*^)	122 (5)	200 (12)	204 (8)	184 (6)	710 (31)
**Boys (%)**	Wealthy districts	50.2	40.2	47.6	48.3	45.8
	Less wealthy districts	50.8	47.5	53.9	51.1	50.8
**Weight (kg)**	Wealthy districts	42 ± 10	46 ± 10	50 ± 10	51 ± 12	47 ± 11
	Less wealthy districts	39 ± 9	43 ± 9	46 ± 10	48 ± 9	45 ± 10
**Height (cm)**	Wealthy districts	146 ± 7	152 ± 7	156 ± 7	158 ± 8	153 ± 8
	Less wealthy districts	144 ± 7	151 ± 7	155 ± 7	158 ± 8	153 ± 9
**BMI**	Wealthy districts	19.5 ± 3.6	19.7 ± 3.2	20.3 ± 3.6	20.0 ± 3.5	19.9 ± 3.5
**(kg/m**^**2**^**)**	Less wealthy districts	18.5 ± 3.4	18.9 ± 3.1	19.2 ± 3.2	19.2 ± 3.0	19.0 ± 3.1
**BMI z-score**	Wealthy districts	0.56 ± 1.32	0.39 ± 1.16	0.28 ± 1.25	−0.11 ± 1.20	0.30 ± 1.25
	Less wealthy districts	0.15 ± 1.38	0.09 ± 1.23	0.12 ± 1.23	−0.41 ± 1.14	−0.09 ± 1.25

*BMI z-score: z-score based on WHO 2007 reference values*[17]. n*: number of missing data.

The overall prevalence of overweight and obesity were 17.8% and 3.2%, respectively (Table 3). As shown in Table 3, the prevalence of overweight and obesity was significantly higher in boys than in girls (p<0.001).

**Table 3:** Prevalence of overweight and obesity among adolescents by age, gender, and puberty stage

**Charateristics**	^**a**^**Overweight**		^**b**^**Obesity**	
	**%**	**PR (95% CI)**	**%**	**PR (95% CI)**
**Gender**				
Boys	22.0	**1.65(1.42, 2.10)**	5.4	**4.15(2.31, 7.74)**
Girls	13.3	**1**	1.3	**1**
**Age**				
11 years	23.6	**2.23(1.59, 3.10)**	4.8	2.18 (1.00, 4.82)
12 years	18.4	**1.74(1.24, 2.42)**	2.8	1.27 (0.59, 3.00)
13 years	18.5	**1.75(1.26, 2.40)**	3.3	1.50 (0.69, 3.22)
≥ 14 years	10.6	**1**	2.2	**1**
**Pubertal stage**				
Pre-pubertal	21.0	1.21 ( 0.94, 1.55)	5.5	**1.90 (1.07, 3.33)**
Pubertal	17.3	**1**	2.9	**1**
**Total**	17.8		3.2	

^*a,b*^*IOTF’s definition for overweight and obesity in adolescents*[15]*. PR: Prevalence ratio.*

Across age groups, there was a decreasing trend with age in overweight and obesity but this trend was only significant for overweight (p<0.001).

Pupils who had reached puberty had lower prevalence of overweight and obesity than those who had not yet reached puberty although the differences were only significant for obesity (p<0.05).

**Figure 1:**
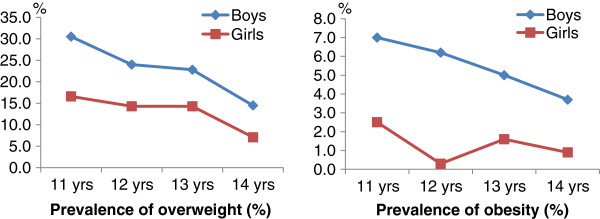
Prevalence of overweight and obesity by age and sex of teenagers from HCMC.

Figure 1 shows the age-and-sex adjusted BMI categories using IOTF cutoffs. Prevalence of overweight decreased significantly as age increased for boys and for girls (p<0.007 for boys and p<0.001 for girls). There was also a decreasing trend in obesity with age for both genders but it was not significant.

Table 4 shows the association between overweight, obesity and socio-economic indicators. As expected, the prevalence of overweight and obesity was lower in pupils studying in schools located in less wealthy districts and in poor families. These differences, however, were only significant for overweight (p<0.001).

**Table 4:** Prevalence of overweight and obesity among adolescents by gender, school location, and economic status of family

**Characteristics**	**Overweight**		**Obesity**	
	**%**	**PR (95% CI)**	**%**	**PR (95% CI)**
**School location**				
Wealthy districts	20.6	**1.70 (1.35, 2.13)**	3.8	1.81 (1.0, 3.15)
Less wealthy districts	12.1	**1**	2.1	**1**
**Household wealthy index**				
Poorest (1st quintile)	12.3	**1**	3.6	**1.0**
2nd quintile	11.7	0.95 (0.65, 1.39)	1.3	0.36 (0.13, 0.99)
3rd quintile	19.9	**1.62 (1.18, 2.27)**	1.9	0.53 (0.22, 1.22)
4th quintile	20.8	**1.69 (1.23, 2.41)**	5.3	1.47 (0.77, 3.00)
Richest (5th quintile)	24.4	**1.98 (1.46, 2.76)**	4.3	1.19 (0.62, 2.47)

*PR: Prevalence ratio.*^*a,b*^*IOTF definition for overweight and obesity in adolescents*[15]*.*

**Figure 2:**
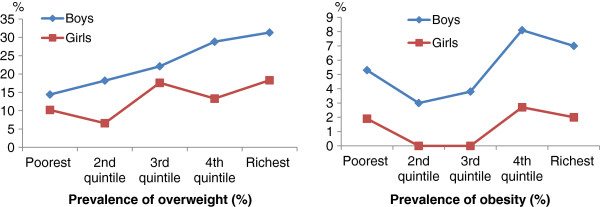
Prevalence of overweight and obesity by economic level and sex of teenagers from HCMC.

Figure 2 illustrates trends of overweight and obesity across economic levels. There was an increasing trend in overweight and obesity from poorest to richest families and the trend was significant for overweight (p<0.001). Again, prevalence of overweight and obesity was higher in boys than in girls within each quintile of economic status.

## Discussion

Our study reports the most recent prevalence of overweight and obesity among adolescents in Ho Chi Minh City. The overall prevalences of overweight and obesity in this representative sample of secondary high schools students of urban areas in Ho Chi Minh City were 17.8% and 3.2%, respectively. Compared to the results of the survey conducted in 2004 in Ho Chi Minh City, which showed a prevalence of 11.7% and 2.1%, respectively [9], our finding indicates a continuing increase of overweight and obesity prevalence.

These prevalences depend upon the IOTF definition. Still today, age- and sex-specific BMI cutoffs for Asian children that are linked to the adult accepted BMI of 23 and 27.5 kg/m^2^ for defining of overweight and obesity do not exist. It is important to establish references in populations of Asian children. The IOTF cutoffs were developed using several data sets and are therefore an international references that can be used to compare world-wide populations which is useful for epidemiology research [18]. These cutoffs were used for defining overweight and obesity among adolescents (11–14 years old) in our study. However, it should be kept in mind that the IOTF BMI cutoffs may not be appropriate for Asian adolescent populations as these cutoffs are mainly based on Caucasian populations; lower cut offs have been reported as more appropriate for defining BMI-related health risk in Asian populations [19]. Using IOTF cutoffs could result in underestimating the overweight and obesity prevalence among Vietnamese adolescents. The CDC 2000 or WHO 2007 definitions were two alternatives. The CDC 2000 growth reference defines children as at risk of overweight and obesity if their BMI exceeds the 85^th^ and 95^th^ centiles [20]. The CDC 2000 reference population is based on USA data, and covered the age range of 2–20 years. The WHO 2007 growth reference defines children as at risk of overweight and obesity if their BMI exceeds +1SD and +2SD or, equivalently, if their BMI z-score exceeds +1 and +2. The WHO 2007 reference population was derived from a combination of Brazil, Ghana, Norway, India, Oman, and USA growth data, and covered the age range of 5–19 years [17]. Prevalences based on these 3 definitions are presented in Table 5. For overweight, IOTF cutoffs resulted in higher prevalences than when using CDC cutoffs in all age- and sex categories, in higher prevalences than when using WHO cutoffs in boys, and in lower prevalences than when using WHO cutoffs in girls. For obesity, IOTF cutoffs resulted in lower prevalences than when using CDC or WHO cutoffs in all age- and sex categories. Such comparisons suggest that the actual prevalences of overweight and obesity are higher than our estimates in adolescents of urban areas of HCMC; the public health problem might be more severe than what is pictured by our data.

**Table 5:** Prevalence (%) of overweight and obesity in boys and girls of HCMC in 2010, using different definitions

**Age (yrs)**	**Boys**				**Girls**				**Total**
	**11**	**12**	**13**	**14**	**11**	**12**	**13**	**14**	
**Overweight (%)**									
IOTF	30.5	24.0	22.8	14.5	16.6	14.3	14.4	7.1	17.8
CDC	21.5	17.5	15.7	9.8	15.0	13.3	14.0	5.8	14.1
WHO	28.2	24.8	20.0	14.1	22.4	19.0	19.0	10.0	19.6
**Obesity (%)**									
IOTF	7.0	6.2	5.0	3.7	2.5	0.4	1.6	0.9	3.2
CDC	18.1	13.0	12.1	7.8	4.2	1.4	2.2	2.3	7.2
WHO	21.6	14.3	12.8	7.2	4.7	2.1	2.2	1.8	7.9

*IOTF: International Obesity Taskforce*[15]*. CDC: The Centers for Disease Control*[20]*. WHO: World Health Organization*[17]*.*

There are some difficulties when trying to compare our findings in Ho Chi Minh City to the results in other countries because the estimates of prevalence of overweight and obesity are based on different reference data, different cutoff values or different age groups. Using the same IOTF criteria, a cross-sectional study conducted in Thailand in 2007 reported prevalences of overweight and obesity in school children (6–15 years old) of 12.8% and 9.4%, respectively [21]. Another cross-sectional study conducted in the northern region of Malaysia among secondary high school children (11–15 years) published in 2006, reported a prevalence of 19.4% for overweight in urban areas, using the WHO definition of a sex- and age-BMI > 85^th^ percentile [22], quite similar to our findings.

The results show that there is a two- to fourfold increase in prevalence for overweight and obesity when comparing boys and girls. From the poorest to the richest households, boys have a higher prevalence of overweight and obesity than girls. In other studies using IOTF cutoff values conducted in Ho Chi Minh City [23], Thailand [24], and Greece [25], similar findings were observed. This difference could be explained by a higher attention to physical appearance among teenage girls as teenage girls fear obesity more than boys.

Another explanation stems from the mentality of male-supremacy in Vietnam. Vietnamese families with sons usually want to feed them as much as possible. There are also differences in physical activities and eating habits between boys and girls [26,27].

Another interesting point to note is a decrease in prevalence of overweight and obesity as age increases, in boys as well as in girls. This trend was also observed in a study conducted in Italy in 2006 [28], and in another study conducted in Singapore [29]. Older teenagers are less dependent of their parents for daily activities; as results they are free to do more activities outside their home or school. In addition, dietary habits of younger children are more dependent on parental control and parents are more likely to encourage younger children to eat more weight and height gain [26].

Our study shows that the prevalences of overweight and obesity are almost doubled in wealthy districts. Such findings are similar to the survey conducted in 2004 [23]. In Vietnam, especially in Ho Chi Minh City, most schools with a high academic standard are located in the wealthy urban areas. Children in affluent families are often sent to these high-standard educational institutions with a high consideration for academic achievement, even if it is detrimental for their health in terms of physical activity or other activities [26].

Children living in wealthier families are more likely to be overweight and obese than those living in less wealthy families. These findings are similar to other studies conducted in developing countries [30] but quite different from industrialized countries where overweight and obesity are more frequent in lower socioeconomic status [31]. In developing countries, obesity is associated with higher socioeconomic status [2]. It is well accepted today that physical inactivity is a major determinant of obesity [32-34]. Wealthier Vietnamese families have a comfortable lifestyle at home, including numerous household electronic appliances that reduce the level of physical activity. Additionally, children in wealthier families are well equipped with modern recreational amenities such as computers, internet, TVs. They therefore spend a lot of time doing sedentary leisure. A recent study among Vietnamese junior high schools students showed that 24.3% of students from junior high schools were inactive [27].

## Conclusions

Our study reports an important emerging public health problem of overweight and obesity in Ho Chi Minh adolescents. Overweight and obesity prevalences continue to increase among secondary-school students. There is therefore an urgent need to setup more aggressive intervention programs targeting good diet habits and physical activities for adolescents in Ho Chi Minh City.

## Abbreviations

BMI: Body mass index;HCMC: Ho Chi Minh City;IOTF: International obesity task force;WHO: World health organization

## Competing interests

None of the authors of the above manuscripts has declared any conflict of interest statement.

## Authors’ contribution

NNVP participated in the design, carried out the study, performed the statistical analysis and drafted the manuscript. TKH provided advice in the design of the study and the analytical strategy and contributed to the manuscript revision. HT helped in the data analysis and report. AR and NTD are head of the project, provided advice about the structure, data analysis and presentation, and supervised the manuscript redaction. All authors read and approved the final manuscript. No author has any financial or private interest in this research project. No organization sponsors this research granted by the University Development Cooperation (http://www.cud.be), which is a Belgian public governmental funding. The corresponding author has full access to all the data in the study and has final responsibility for the decision to submit for publication.

## Pre-publication history

The pre-publication history for this paper can be accessed here:

http://www.biomedcentral.com/1471-2458/13/141/prepub
